# Single-Organ Vasculitis Following COVID-19 Infection Resulting in Visceral Arterial Rupture: A Case Report

**DOI:** 10.7759/cureus.87335

**Published:** 2025-07-05

**Authors:** Yasutaka Saito, Shinichiro Irabu, Hirotaka Yamamoto

**Affiliations:** 1 Department of Hepatobiliary and Pancreatic Surgery/Acute Care Surgery, Seirei Hamamatsu General Hospital, Hamamatsu, JPN

**Keywords:** covid-19, covid-19-associated vasculitis, gastroepiploic artery rupture, intraperitoneal hemorrhage, single organ vasculitis

## Abstract

We present the case of a male in his 50s who developed sudden abdominal pain two days after testing positive for coronavirus disease 2019 (COVID-19) and was brought to the emergency department in shock. An emergency laparotomy revealed intraperitoneal hemorrhage due to spontaneous rupture of the right gastroepiploic artery. Histopathological examination revealed necrotizing vasculitis, while systemic causes were excluded. The case was diagnosed as abdominal single-organ vasculitis (SOV) likely triggered by COVID-19. This rare presentation highlights the importance of considering SOV in COVID-19 patients presenting with acute abdomen, as early recognition and intervention are critical to avoid life-threatening hemorrhage.

## Introduction

Vasculitis is a known complication of coronavirus disease 2019 (COVID-19), and its manifestations range from leukocytoclastic vasculitis and IgA vasculitis to Kawasaki-like syndromes [1]. While systemic involvement has been frequently described, isolated single-organ vasculitis (SOV) has been infrequently reported [2]. SOV is defined as “vasculitis confined to a single organ without features of systemic vasculitis”, according to the 2012 revised Chapel Hill Consensus Conference nomenclature [3]. Although rare, SOV has been associated with infections, malignancy, and medications.

COVID-19-related SOV most commonly affects the skin, kidneys, and lungs, whereas involvement of visceral organs, such as the gastrointestinal tract, is exceedingly rare. Abdominal SOV may present with nonspecific symptoms such as localized pain or intraperitoneal bleeding, making early diagnosis challenging. While a few cases have been reported in the literature, none have been definitively diagnosed, particularly with histopathological confirmation. We report a rare case of COVID-19-associated abdominal SOV leading to a spontaneous rupture of the right gastroepiploic artery, diagnosed histopathologically following surgical intervention.

## Case presentation

A 52-year-old male with a history of hypertension but no medications developed COVID-19 symptoms and tested positive for the same. Two days later, he experienced sudden, severe abdominal pain with diaphoresis and was transported to our emergency department. On arrival, the patient was in shock, presenting with pallor, hypotension, tachycardia, and tachypnea. Radial pulses were impalpable, and body temperature was 36.2 °C. Arterial blood gas analysis indicated metabolic acidosis, and laboratory tests showed mild anemia, elevated inflammatory markers, and coagulopathy (Table 1).

**Table 1:** Laboratory values on admission The results indicate metabolic acidosis, mild anemia, systemic inflammation, and coagulation abnormalities APTT: activated partial thromboplastin time; CRP: C-reactive protein; PT-INR: prothrombin time-international normalized ratio

Parameter	Patient value	Reference range		Interpretation
Hemoglobin, g/dL	9.9	13.0–17.0		Decreased
CRP, mg/L	22.1	<5.0		Increased
PT-INR	1.13	0.85–1.15		Normal
APTT, seconds	30.7	25.0–35.0		Normal
Fibrinogen, mg/dL	238	200–400		Normal
D-dimer, μg/dL	9.9	<1.0		Increased
Arterial pH	7.09	7.35–7.45		Decreased
Base excess, mEq/L	-14.8	-2 to +2		Decreased
Lactate, mg/dL	123	<18.0		Increased

Imaging

**Figure 1:**
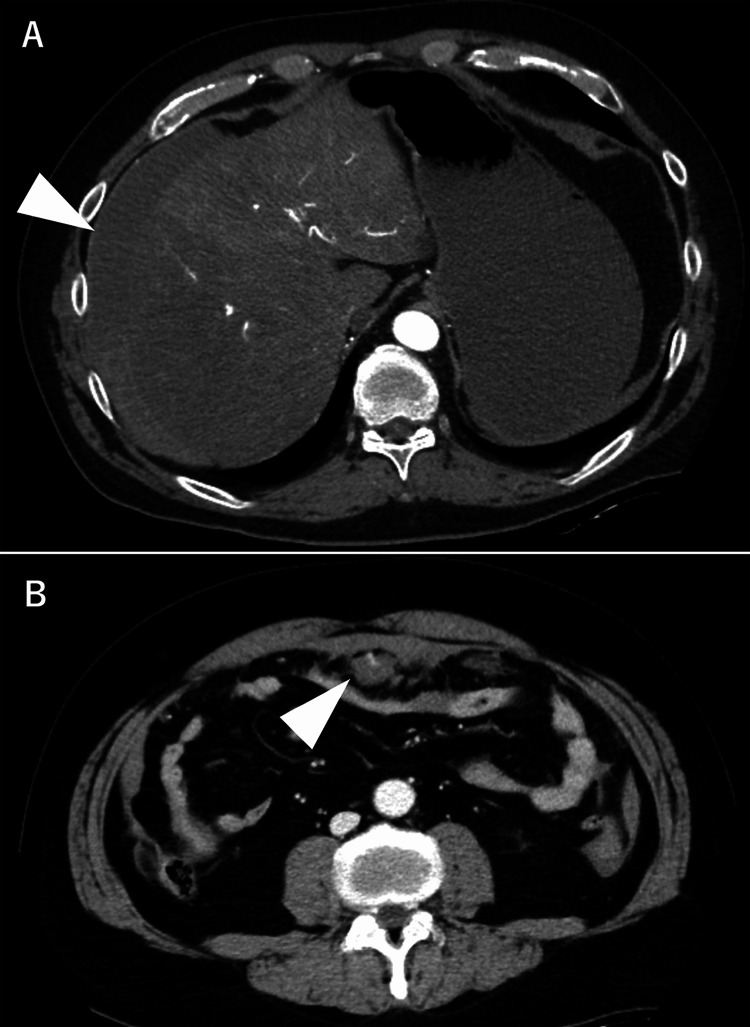
Contrast-enhanced CT findings on day 1 A. Contrast-enhanced CT at admission showing hemoperitoneum on the liver surface(arrow). B. Contrast-enhanced CT revealing a hematoma in the fat tissue with contrast extravasation inside (arrow) CT: computed tomography

Ultrasound suggested intra-abdominal bleeding, prompting an immediate transfusion [four units each of red blood cells (RBCs) and fresh frozen plasma (FFP)] and contrast-enhanced CT. CT revealed a hemoperitoneum and hematoma in the upper abdominal fat tissue near the umbilicus, with suspected contrast extravasation (Figures 1A, 1B). No vascular malformation or aneurysm was observed*. *Despite receiving a transfusion, the patient remained hemodynamically unstable, necessitating an emergency exploratory laparotomy.

Intraoperative findings

**Figure 2:**
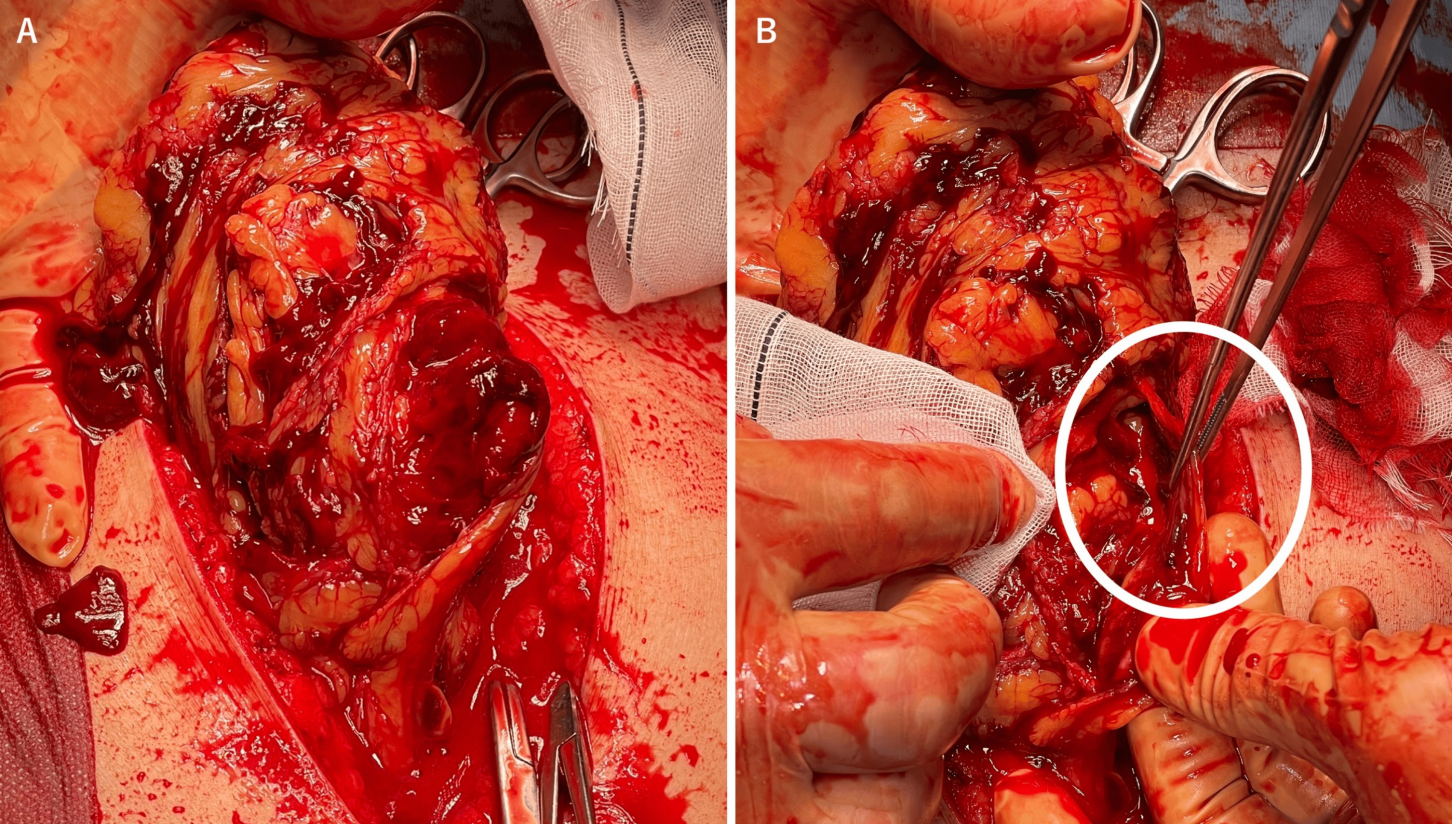
Intraoperative findings A. Extensive contusion of the greater omentum and hematoma within the omentum. B. Arterial bleeding from the rupture site of the right gastroepiploic artery (circled)

A large volume of intraperitoneal hemorrhage was observed. A hematoma and active bleeding were identified in the greater omentum. The bleeding source was the right gastroepiploic artery (Figures 2A, 2B). The ruptured vessel was resected, along with the omentum damaged by the hematoma. No other lesions were found on inspection of the gastrointestinal tract and mesentery. The patient was admitted to the ICU for ventilatory support. The estimated blood loss was 2,261 mL; the total transfusion was 14 units of RBCs and eight units of FFP.

Pathology

**Figure 3:**
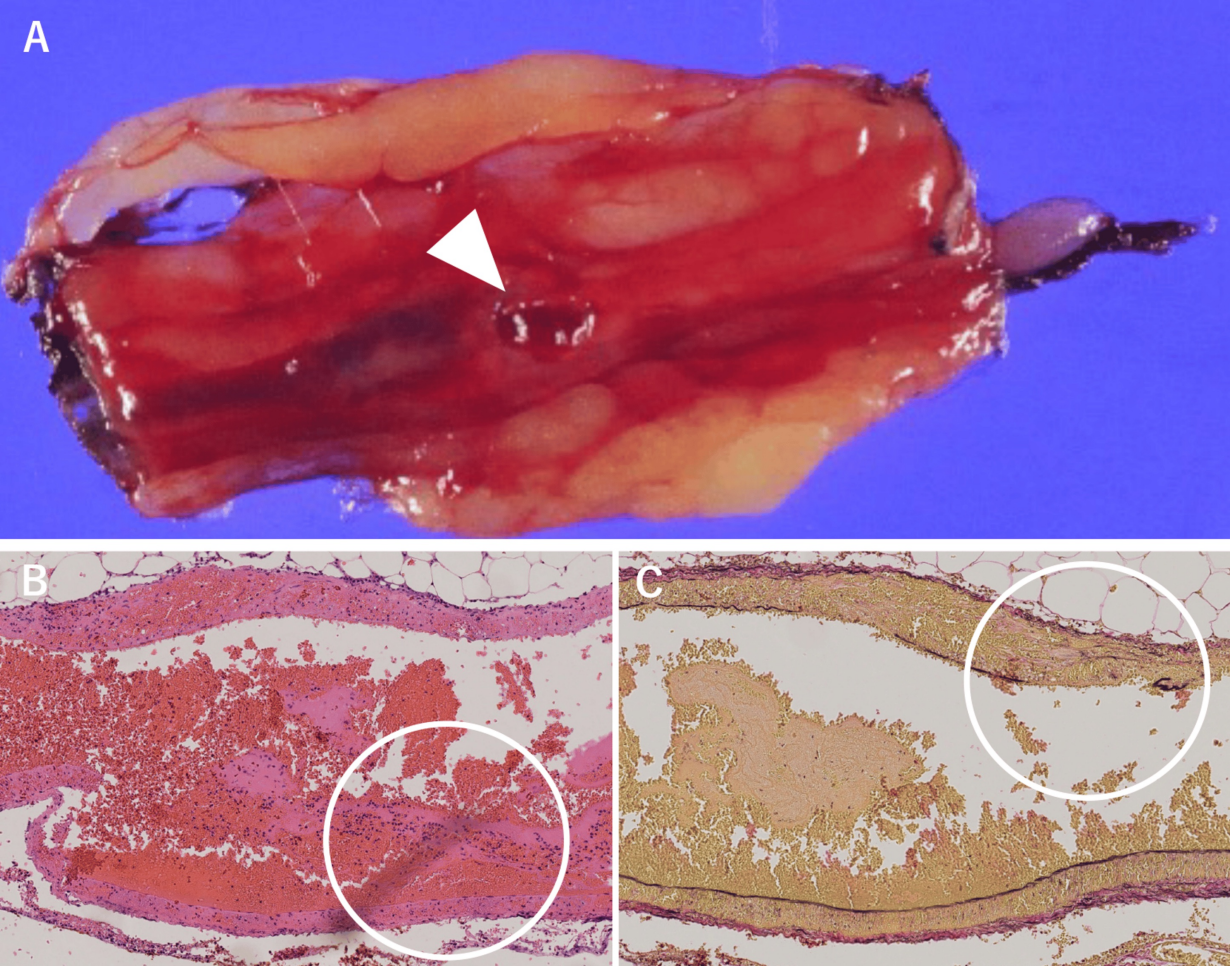
Pathological findings A. The vessels and ruptured artery within the omentum (arrow). B. These vessels show transmural fibrinoid necrosis and marked neutrophilic infiltration on HE staining (circled). C. Elastic fiber fragmentation was observed on EVG staining, consistent with necrotizing vasculitis (circled) EVG: Elastica van Gieson; HE: hematoxylin–eosin

A histological examination revealed transmural fibrinoid necrosis and dense neutrophilic infiltration. Elastic staining showed disruption of the elastic lamina, consistent with necrotizing vasculitis (Figures 3A, 3B, 3C).

Postoperative course

The patient was extubated on postoperative day one and discharged home on day eight without any complications. No additional treatment for COVID-19 was required, and there has been no recurrence. Autoimmune and systemic vasculitis screenings were negative, including anti-nuclear antibody (ANA), anti-neutrophil cytoplasmic antibody (ANCA) [myeloperoxidase (MPO), proteinase 3 (PR3)], complement levels, cryoglobulins, and antiphospholipid antibodies.

## Discussion

COVID-19, caused by severe acute respiratory syndrome coronavirus 2 (SARS‑CoV‑2), has been associated with the development of various vasculitides. Even in mild cases, localized vascular endothelial injury has been reported, contributing to SOV manifestations, including deep vein thromboses and cutaneous vasculitis [4]. SARS-CoV-2 has been shown to invade endothelial cells via the angiotensin-converting enzyme 2 (ACE2) receptor, causing vascular inflammation and hemorrhage [5]. Previous reports of abdominal SOV in COVID-19 patients include a few suspected cases [6-8], some involving intra-abdominal bleeding and surgical intervention [6]. However, none of those cases were histopathologically confirmed. In our case, systemic vasculitis and autoimmune disease were ruled out, and the diagnosis of COVID-19-associated SOV was confirmed by histopathological exam.

SOV has been reported in various organs throughout the body and is often associated with infections, medications, and malignancies, though it is frequently diagnosed incidentally. Exclusion of systemic vasculitis is essential for establishing the diagnosis. Gastrointestinal SOV is less common than gastrointestinal involvement in systemic vasculitis, particularly polyarteritis nodosa (PAN) [9]. SOV has been reported in the appendix (the most common site), esophagus, stomach, omentum, small intestine, colon, gallbladder, and pancreas. As in our patient, abdominal pain is the most common presenting symptom. In general, SOV has a favorable prognosis and rarely progresses to systemic vasculitis. Gastrointestinal SOV, however, shows a more aggressive behavior with complications such as ulceration, bowel perforation, peritonitis, hemorrhage, and intestinal ischemia, with reported mortality rates reaching up to 40%. Also, progression to systemic vasculitis has been reported in up to 26% of gastrointestinal SOV cases [10]. Therefore, in gastrointestinal SOV cases where medical (non-surgical) management is selected, careful surveillance is warranted.

Currently, no standardized treatment protocol exists for SOV. In many cases, resection of the affected lesion leads to complete resolution. In cases of secondary vasculitis, treatment should target the underlying disease, and, depending on the degree of inflammation or symptoms, the addition of corticosteroids or immunosuppressive agents may be appropriate [11]. Corticosteroids are typically considered the first-line treatment for vasculitis in the presence of systemic involvement or organ dysfunction. In gastrointestinal SOV, clinical signs such as persistent abdominal pain, fever, elevated inflammatory markers, or worsening imaging findings may suggest ongoing vasculitic activity and warrant immunosuppressive therapy in addition to surgical intervention. However, treatment decisions should be individualized based on the clinical course, histopathological severity, and presence of systemic inflammation.

When vascular rupture due to SOV necessitates surgical intervention, the appropriateness of adjunctive therapies such as corticosteroids depends on the extent of vasculitic involvement, histopathological findings, and systemic inflammatory activity. While COVID-19-associated systemic vasculitis has been reported primarily in severe cases [12], the relationship between COVID-19 severity and treatment response in abdominal SOV remains unclear. Although our patient initially presented with mild upper respiratory symptoms consistent with COVID-19, he did not develop systemic or respiratory complications during hospitalization. The decision to forego additional treatment was not based on the severity of COVID-19, but rather on the localized nature of the vasculitis and the uneventful postoperative course. The patient exhibited no abdominal symptoms, and inflammatory markers normalized without any evidence of systemic progression.

Early diagnosis of abdominal SOV is often challenging due to its nonspecific symptoms. However, in patients with a recent or active history of COVID-19 who present with acute abdominal pain, the presence of unexplained intraperitoneal hemorrhage, localized peritonitis, or elevated inflammatory markers should prompt consideration of this condition. Ultrasonography and contrast-enhanced CT may reveal inflammatory changes in the vasculature, and in more severe cases, signs of bowel ischemia, perforation, or hemorrhage. Prompt recognition and diagnosis based on these clinical and radiological findings may facilitate timely intervention and contribute to improved outcomes.

Given the risk of life-threatening complications such as bowel perforation or hemorrhage in gastrointestinal SOV, active treatment, including surgical intervention and supportive care, should be strongly considered even in cases with mild symptoms. Early recognition and timely management are essential to prevent catastrophic outcomes. This report underscores the importance of histopathological confirmation in clarifying the etiology of abdominal SOV, and further accumulation of similar reports will help establish more precise diagnostic and therapeutic strategies.

## Conclusions

We presented a rare case of abdominal single-organ vasculitis associated with COVID-19 that resulted in a spontaneous rupture of the right gastroepiploic artery. This condition can lead to a life-threatening hemorrhage. SOV should be considered in the differential diagnosis of abdominal pain in COVID-19 patients. Given the risk of vascular rupture, prompt diagnosis and rapid access to emergency surgical care are essential.
